# Supplementary material for: Protective Effect and Mechanism of Boswellic Acid and Myrrha Sesquiterpenes with Different Proportions of Compatibility on Neuroinflammation by LPS-Induced BV2 Cells Combined with Network Pharmacology
Source: Molecules. 2019 Oct 31;24(21):3946. doi: 10.3390/molecules24213946 (PMC6864549; doi:10.3390/molecules24213946)
Supplement: Supplementary file 1 [file molecules-24-03946-s001.pdf]

Figure.S1 the ion flow chromatogram of the mixed reference material on the left and the ion flow chromatogram of the sample on the right.

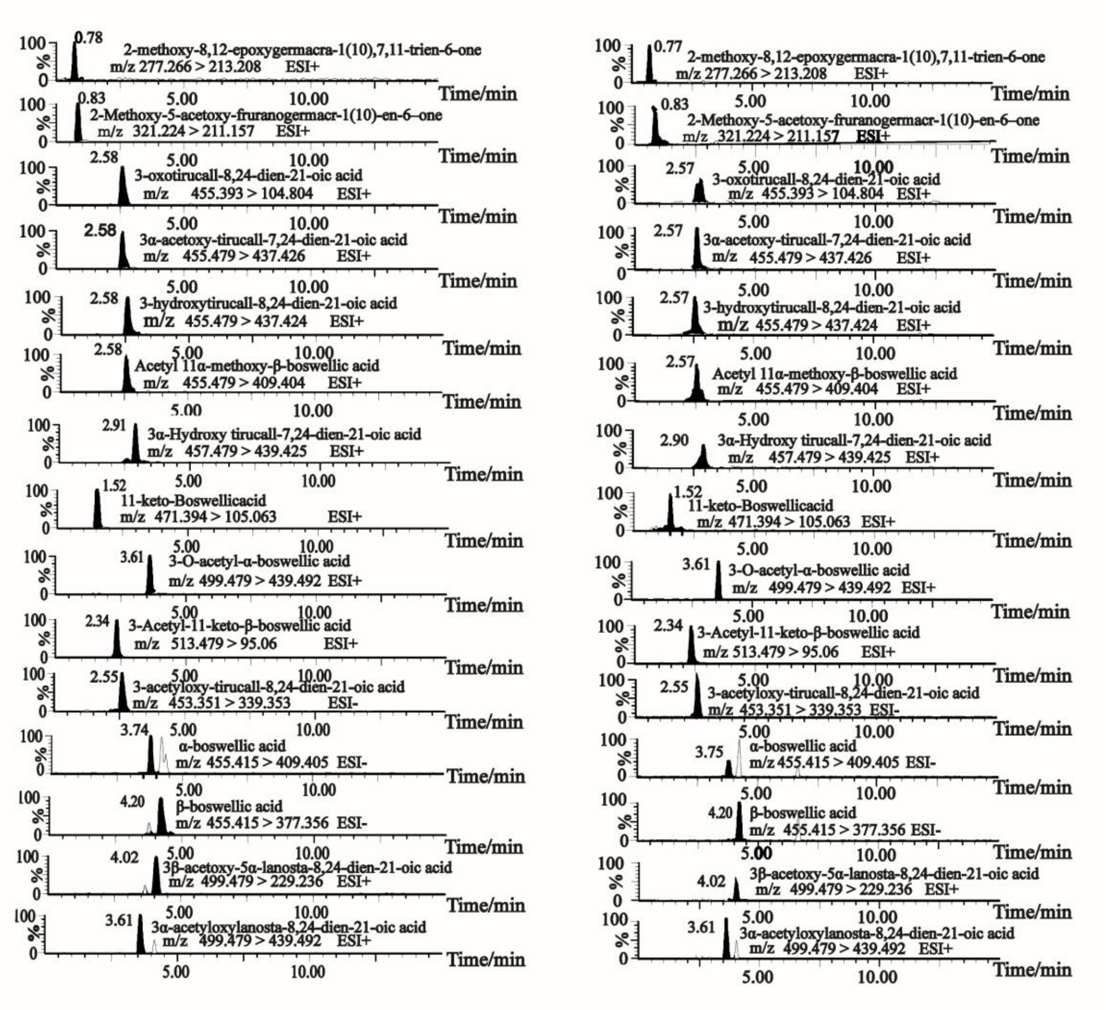

Table.S1 MRM transition， conevoltage， collision energy and ion mode of 15 investigated compounds

(NO.)	compound	MW	(MRM transition)m/z	(cone voltage)/V	(collision energy)/ eV	(ion mode)
1	2-methoxy-8,12-epoxygermacra-1(10),7,11-trien-6-one	277	277.266→213.208	8	14	ESI^+
2	2-methoxy-5-acetoxy-fruranogermacr-1(10)-en-6–one	320	321.224→229.17	12	12	ESI^+
3	3-oxotirucall-8,24-dien-21-oic acid	453	453.393→104.804	70	32	ESI^+
4	3α-acetoxy-tirucall-7,24-dien-21-oic acid	454	455.479→437.426	14	8	ESI^+
5	3-hydroxytirucall-8,24-dien-21-oic acid	454	455.479→437.424	14	14	ESI^+
6	acetyl 11α-methoxy-β-boswellic acid	454	455.479→409.404	16	22	ESI^+
7	3α-hydroxy tirucall-7,24-dien-21-oic acid	456	457.479→439.425	18	12	ESI^+
8	11-keto-boswellic acid	471	471.394→105.063	60	36	ESI^+

9	3-O-acetyl-α-boswellic acid	498	499.479→439.492	55	22	ESI^+
10	3α-acetyloxylanosta-8,24-dien-21-oic acid	498	499.479→439.492	54	34	ESI^+
11	3β-acetoxy-5α-lanosta-8,24-dien-21-oic acid	498	499.479→229.236	54	32	ESI^+
12	3-acetyl-11-keto-β-boswellic acid	512	513.479 → 95.06	40	42	ESI^+
13	3-acetyloxy-tirucall-8,24-dien-21-oic acid	454	453.354→339.353	70	30	ESI^-
14	α-boswellic acid	*456*	455.415→409.405	50	22	ESI^-
15	β-boswellic acid	*456*	455.415→377.356	44	30	ESI^-

Table.S2 Linear regression data

NO.	compound	regression equation	*r*	（linear range)/ μg/mL
1	2-methoxy-8,12-epoxygermacra-1(10),7,11-trien-6-one	$y = 0.3588x - 0.2075$	0.9889	0.0890~11.40
2	2-methoxy-5-acetoxy-fruranogermacr-1(10)-en-6–one	$y = 93.028x + 24.451$	0.9982	0.1750~11.30
3	3-oxotirucall-8,24-dien-21-oic acid	$y = 91.974x + 33.939$	0.9995	0.4090~13.10
4	3*α*-acetoxy-tirucall-7,24-dien-21-oic acid	$y = 209.62x + 113.46$	0.9974	0.1328~17.00
5	3-hydroxytirucall-8,24-dien-21-oic acid	$y = 497.05x + 85.839$	0.9909	0.3187~20.40
6	acetyl 11*α*-methoxy-*β*-boswellic acid	$y = 57.58x + 22.848$	0.9957	0.2070~13.25
7	3*α*-hydroxy tirucall-7,24-dien-21-oic acid	$y = 78.189x - 23.028$	0.9999	0.6062~38.80
8	11-keto-boswellicacid	$y = 1128.9x + 319.09$	0.9935	0.0203~20.80
9	3-*O*-acetyl-*α*-boswellic acid	$y= 3.4072x - 1.536$	0.9911	0.8312~53.20
10	3*α*-acetyloxylanosta-8,24-dien-21-oic acid	$y= 26.375x + 17.669$	0.9904	0.1719~44.00
11	3*β*-acetoxy-5*α*-lanosta-8,24-dien-21-oic acid	$y= 57.399x - 36.62$	0.9984	1.619~12.95
12	3-acetyl-11-keto-*β*-boswellic acid	$y= 851.08x + 294.9$	0.9962	0.7187~23.60
13	3-acetyloxy-tirucall-8,24-dien-21-oic acid	$y= 1000.6x + 2582.6$	0.9921	0.2656~34.00
14	*α*-boswellicacid	$y= 5.4652x - 6.311$	0.9968	0.0804~41.20
15	*β*-boswellicacid	$y= 11.778x + 46.189$	0.9906	0.7875~50.40
